# MicroRNA–Messenger RNA Regulatory Network Mediates Disrupted TH17 Cell Differentiation in Depression

**DOI:** 10.3389/fpsyt.2022.824209

**Published:** 2022-04-05

**Authors:** Haiyang Wang, Lanxiang Liu, Xueyi Chen, Chanjuan Zhou, Xuechen Rao, Wenxia Li, Wenwen Li, Yiyun Liu, Liang Fang, Hongmei Zhang, Jinlin Song, Ping Ji, Peng Xie

**Affiliations:** ^1^Key Laboratory of Psychoseomadsy, Stomatological Hospital of Chongqing Medical University, Chongqing, China; ^2^College of Stomatology and Affiliated Stomatological Hospital of Chongqing Medical University, Chongqing, China; ^3^Chongqing Key Laboratory for Oral Diseases and Biomedical Sciences, Chongqing, China; ^4^National Health Commission Key Laboratory of Diagnosis and Treatment on Brain Functional Diseases, The First Affiliated Hospital of Chongqing Medical University, Chongqing, China; ^5^Department of Neurology, Yongchuan Hospital of Chongqing Medical University, Chongqing, China; ^6^Department of Pathology, Faculty of Basic Medicine, Chongqing Medical University, Chongqing, China

**Keywords:** depression, miRNAs, mRNAs, Th17 cell differentiation, CSDS

## Abstract

Accumulating evidence indicates an important role for microRNA (miRNA)–messenger RNA (mRNA) regulatory networks in human depression. However, the mechanisms by which these networks act are complex and remain poorly understood. We used data mining to identify differentially expressed miRNAs from GSE81152 and GSE152267 datasets, and differentially expressed mRNAs were identified from the Netherlands Study of Depression and Anxiety, the GlaxoSmithKline-High-Throughput Disease-specific target Identification Program, and the Janssen-Brain Resource Company study. We constructed a miRNA–mRNA regulatory network based on differentially expressed mRNAs that intersected with target genes of differentially expressed miRNAs, and then performed bioinformatics analysis of the network. The key candidate genes were assessed in the prefrontal cortex of chronic social defeat stress (CSDS) depression mice by quantitative real-time polymerase chain reaction (qRT-PCR). Three differentially expressed miRNAs were commonly identified across the two datasets, and 119 intersecting differentially expressed mRNAs were identified. A miRNA–mRNA regulatory network including these three key differentially expressed miRNAs and 119 intersecting differentially expressed mRNAs was constructed. Functional analysis of the intersecting differentially expressed mRNAs revealed that an abnormal inflammatory response characterized by disturbed T-helper cell 17 (Th17) differentiation was the primary altered biological function. qRT-PCR validated the decreased expression of Th17 cell differentiation-related genes, including *interleukin (IL)17A, IL21, IL22*, and *IL1*β, and the increased expression of retinoic acid receptor-related orphan receptor gamma-t (*ROR*γ*t*) in CSDS mice, which showed significant depressive- and anxiety-like behaviors. This study indicates that an abnormal inflammatory response characterized by disturbed Th17 cell differentiation is the primary altered biological process in major depressive disorder. Our findings indicate possible biomarkers and treatment targets and provide novel clues to understand the pathogenesis of major depressive disorder.

## Introduction

Major depressive disorder (MDD) is a debilitating disease and contributes greatly to the global burden of disease (1). One in six adults were reported to be affected by depression, which not only can dramatically affect one's ability to work and function, but also increases the risk of developing other conditions, such as cardiovascular diseases and diabetes mellitus (2). In addition, the comorbidity of depression with other psychiatric disorders (e.g., anxiety) is increasingly prevalent, and this can lead to an increased risk for suicidality (3, 4). However, up to 40% of patients do not remit from MDD, even after several treatment attempts (5). This may be because of the biological heterogeneity of depression (6); therefore, further exploration of the biological processes underlying depression is essential for precision medicine.

Preclinical animal models have enabled the discovery of neurophysiological mechanisms underlying depression (7). We previously verified that different categories of stressor, “physical” and “psychological,” elicit distinctive molecular responses (8). However, the symptoms of human depression can only partly be mimicked in animals. Therefore, biological samples from patients are valuable for ascertaining the molecular mechanisms of depression and for identifying diagnostic biomarkers. Previously, we used a metabolomics approach to uncover molecular changes in MDD patients. We found that acyl carnitine metabolism, lipid metabolism, and tryptophan metabolism were the primary disturbed pathways in adult patients (9, 10), while polyunsaturated fatty acid metabolism and purine metabolism were prominent pathways in child and adolescent patients (11). Moreover, accumulating evidence suggest pivotal roles of inflammatory responses in MDD pathophysiology (12–14). Inflammatory mediators and cytokines regulate immune responses through anti-inflammatory and pro-inflammatory pathways (15, 16). T-cell cytokines play a central role in the initiation and regulation of cellular immune responses (17, 18). The imbalance of different T cells, such as T-helper 17 cells (Th17) and regulatory T cells is critical for the progression of MDD. However, the underlying mechanisms of depression are complex and remain poorly understood.

Non-coding RNAs are essential epigenetic regulators involved in complex biological processes and play key roles in the pathogenesis of MDD. MicroRNAs (miRNAs) are endogenous small non-coding RNAs ~22 nucleotides in length that regulate gene expression by binding to the 3′-untranslated region of target messenger RNAs (mRNAs) (19), which leads to translational repression (20). Many miRNAs are functionally dysregulated in MDD (21). For example, our previous studies indicate dysregulation of various miRNAs (e.g., miR-190a-5p, miR-539-5p, and miR-363-5p) in depression-related brain dysfunction (22). We have also shown downregulation of miR-134 in MDD patients, which may be recognized as a state-dependent biomarker for depression (23). In addition, miR-138 increased depressive-like behaviors in mice by regulating SIRT1 (24). However, the miRNA–mRNA interactions in the pathogenesis of depression need to be further explored.

We hypothesize that the disorganized immune response and the miRNA–mRNA interactions that regulate Th17 cell differentiation are potential therapeutic targets in MDD. In this study, we performed a systematic analysis to identify aberrant miRNAs based on public databases. We then constructed a miRNA–mRNA interaction network and performed bioinformatics functional analysis to predict the potential mechanisms of these miRNA–mRNA pairs in the development of MDD. Furthermore, the MDD-associated candidate genes were assessed in the prefrontal cortex of chronic social defeat stress (CSDS) depression mice by quantitative real-time polymerase chain reaction (qRT-PCR). This study identifies potential diagnostic biomarkers and provides clues for exploring the pathogenesis of MDD.

## Materials and Methods

### miRNA Microarray

We searched for suitable datasets that focused on miRNA changes between patients with MDD and healthy controls in the Gene Expression Omnibus (GEO) database (http://www.ncbi.nlm.nih.gov/geo/). After evaluating the datasets of interest, original miRNA profiles from two datasets (GSE81152 and GSE152267), based on the Exiqon Human miRCURY LNA microRNA Array (GPL21814) and the [miRNA-4] Affymetrix Multispecies miRNA-4 Array (GPL21572), respectively, were obtained for subsequent analysis. The GSE81152 dataset contained 30 pre-treatment MDD patients and 19 healthy controls, and the GSE152267 dataset contained 7 MDD patients and 6 healthy controls.

### Identification of Differentially Expressed miRNAs

GEO2R (http://www.ncbi.nlm.nih.gov/geo/geo2r/), an interactive web tool based on the limma R package, was used to identify the differentially expressed miRNAs (DEMs) between MDD patients and health controls from the two datasets. The DEMs were identified by the thresholds of |log_2_FC| > 1 and *p* < 0.05. The common DEMs identified from both GSE81152 and GSE152267 datasets were re-defined as key miRNAs and used for further analysis. A Venn diagram was produced using the E-Venn online tool (http://www.ehbio.com/test/venn/#/).

### Prediction of DEM Targets

The downstream target genes of DEMs were predicted using TargetScan (25), DIANA-TarBase v8 (26), miRDB (27), miRTarBase (28), and miRWalk (29).

### Construction of a miRNA–mRNA Interaction Network

To identify MDD-related genes, differentially expressed mRNAs (DEGs) were selected from the Netherlands Study of Depression and Anxiety (NESDA) (30), the GlaxoSmithKline-High-Throughput Disease-specific target Identification Program (GSK-HiTDiP), and the Janssen-Brain Resource Company (Janssen-BRC) study (31). The identified DEGs were intersected with target genes of DEMs. The intersected DEG sets were used to construct the miRNA–mRNA network on the basis of the interactions between key DEMs and DEGs, and this regulatory network was visualized using Cytoscape (version 3.7.2) (32).

### Functional Enrichment Analysis

To further assess the biological functions of the miRNA–mRNA pairs, Gene Ontology (GO) annotation, and Kyoto Encyclopedia of Genes and Genomes (KEGG) enrichment pathway analyses were performed for the intersected DEGs using the online OmicsBean system (http://www.omicsbean.cn). *p*-values are calculated using Fisher's Exact test with a hypergeometric algorithm. GO terms, including biological process, cellular component and molecular function, and KEGG pathways were considered to be significantly enriched with a *p* < 0.05.

### Protein–Protein Interaction Analysis

To obtain a better understanding of the relationships among the genes of interest, a PPI network was established based on the Search Tool for the Retrieval of Interacting Genes (STRING) database (https://string-db.org/), and visualized using Cytoscape (version 3.7.2). Any interaction with a combined score ≥0.4 was included in the PPI network.

### CSDS Model of Depression

Adult male C57BL/6 J mice (weighing 20–23 g) and male CD1 mice (weighing 35–40 g) were obtained from the Experimental Animal Center of Chongqing Medical University (Chongqing, China). This experiment was approved by the Ethics Committee of Chongqing Medical University. The mice were kept under standard conditions (constant temperature, 21 ± 1°C; relative humidity, 55 ± 5%; and a 12-h light cycle with lights on at 8:00) with food and water available *ad libitum*. The CSDS procedure was conducted as previously described (33). Briefly, CSDS was initiated when an “intruder” (C57BL/6 J mouse) was introduced into the home cage of an aggressive “resident” (CD1 mouse) for physical contact lasting 10 min. Subsequently, the intruder was immediately transferred to the opposite compartment of the defeated cages, which allowed sensory contact for 24 h. After the 12-day CSDS period, a series of behavioral tests was performed.

A social interaction test was used to detect social avoidance behaviors. The immobility time in the forced swimming test and tail suspension test was used to evaluate depression-like behaviors. Time spent in the central and perimeter areas during the open field test (34), as well as the time spent in the open and closed arms of an elevated plus-maze are recognized as measures of anxiety-like behaviors. The data are expressed as the mean ± standard error of the mean (SEM), and statistical analysis was performed with the Mann–Whitney *U*-test using SPSS 21.0 (IBM, New York, USA).

### RNA Extraction and qRT-PCR

Total RNA was extracted from prefrontal cortex tissues using Trizol reagent (Invitrogen, USA). A total of 1 μg of RNA was reverse transcribed into cDNA using a PrimeScript RT reagent Kit (Takara, Japan). For RNA quantification, three replicate CSDS and control samples were analyzed using the SYBR gene detection system (Roche, Germany). Expression levels of target miRNAs were normalized to small nuclear RNA U6, and those of mRNAs were normalized to β-actin, and calculated using the 2^−ΔΔCt^ method. We used non-parametric statistical methods to reduce the effect of the small sample size. The primers used are shown in Supplementary Table S1.

## Results

### Screening for Significant DEMs in MDD

**Figure 1:**
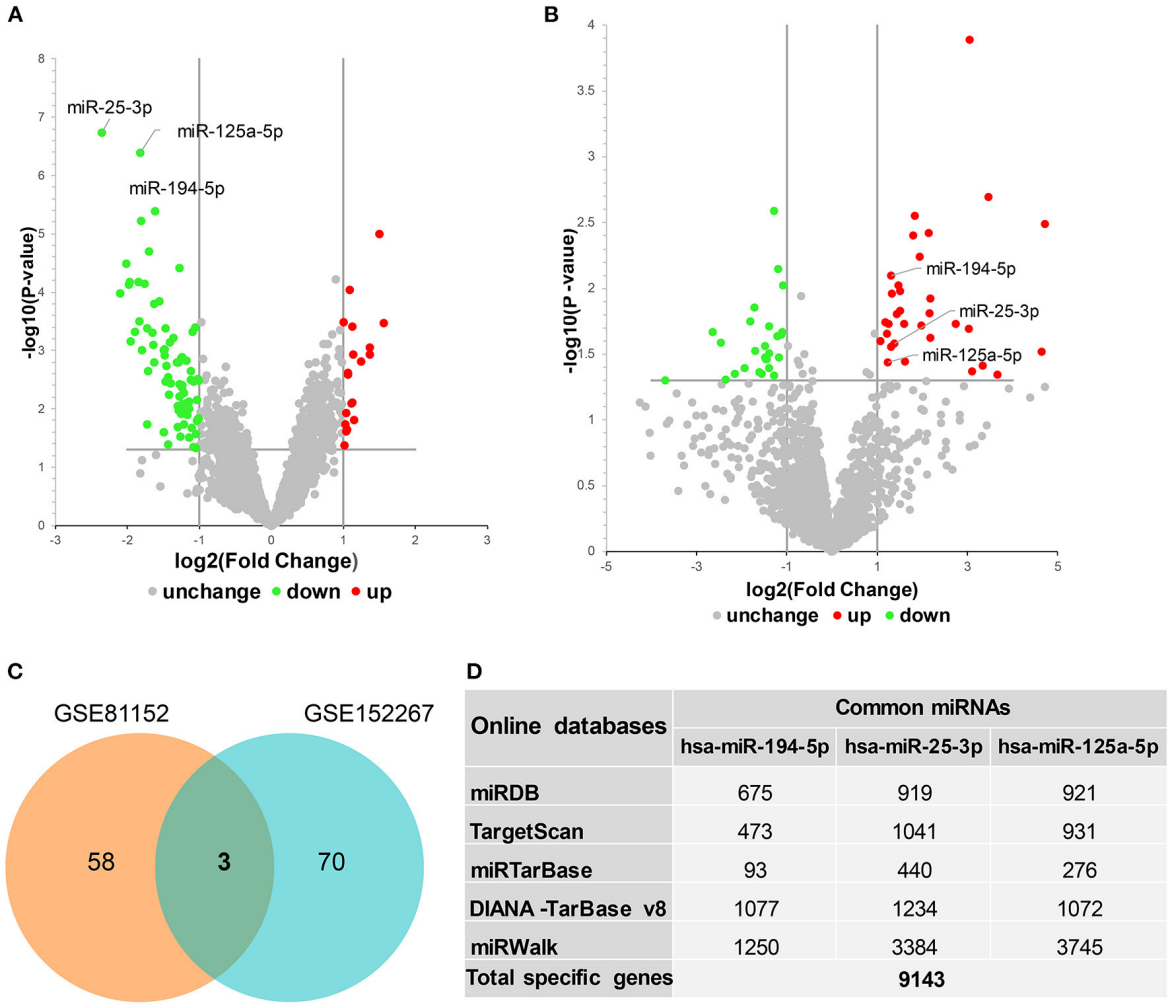
Identification of differentially expressed miRNAs (DEMs) in major depressive disorder. **(A,B)** Volcano plots showing DEMs identified from GSE81152 and GSE152267 datasets, respectively. UP, upregulated; DW, downregulated; NoDiff, no differential expression. **(C)** Venn diagram showing the intersected DEMs between GSE81152 and GSE152267 datasets. **(D)** The prediction of target genes of these common DEMs.

miRNA microarray datasets in the GEO database relevant to MDD were searched for and GSE81152 and GSE152267 datasets were identified. The interactive web tool, GEO2R, was then used to analyze the DEMs from each dataset. Volcano plots showing the DEMs from GSE81152 and GSE152267 datasets are displayed in Figures 1A,B, respectively. We identified 61 DEMs in the GSE81152 dataset and 73 in the GSE152267 dataset. We then analyzed overlap of the DEMs from the two datasets, and found three overlapping DEMs (Figure 1C), hsa-miR-194-5p, hsa-miR-25-3p, and hsa-miR-125a-5p. These common DEMs were selected for further analysis.

### Construction of a miRNA–mRNA Regulatory Network in MDD

**Figure 2:**
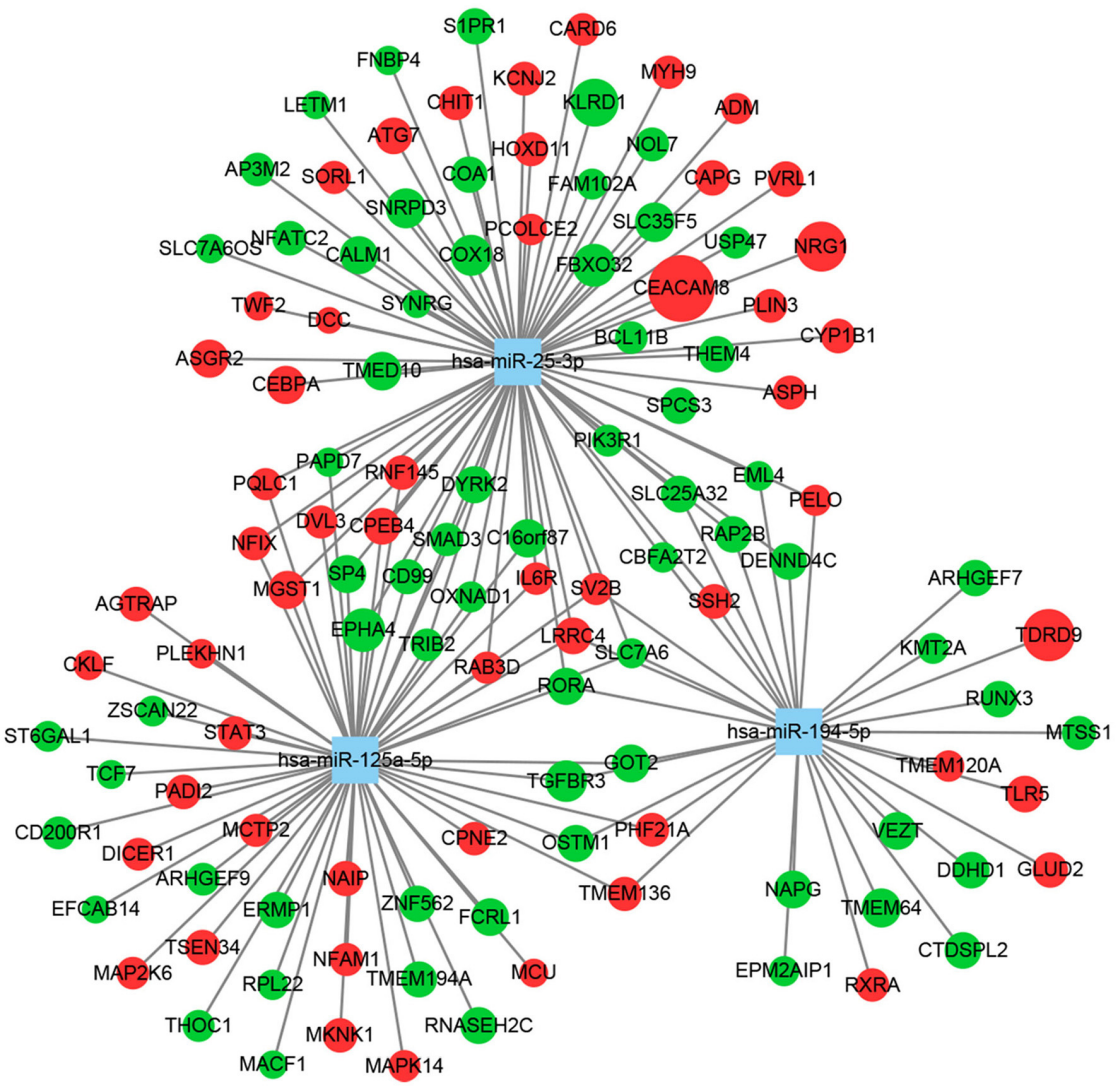
Construction of a miRNA–mRNA regulatory network using Cytoscape. Squares represent miRNAs, circles represent mRNAs, green indicates downregulation, red indicates upregulation, and circle size reflects the degree of change.

We first predicted targets of the miRNAs using several online software packages and identified 9,143 target genes after removing duplicates (Figure 1D). In addition, to analyze changes in MDD at the mRNA level, we reanalyzed case–control studies focusing on gene expression in peripheral blood of MDD patients. As a result, we selected 129 DEGs between the MDD and control groups in the NESDA study, as well as 165 DEGs in both GSK-HiTDiP and Janssen-BRC studies that had concordant direction of expression changes. Merging these identified DEGs with the 9,143 DEM target genes identified 119 intersected DEGs. Finally, we used the three key DEMs and the 119 intersected DEGs to construct a miRNA–mRNA regulatory network based on the interactions between miRNAs and mRNAs (Figure 2). As shown in this miRNA–mRNA interaction network, hsa-miR-25-3p has the most interaction relationships with 70 pairs, followed by hsa-miR-125a-5p with 55 pairs and hsa-miR-194-5p with 31 pairs. Interestingly, several genes were targeted by all three key DEMs, such as *Rora*, indicating the potential roles of these genes in the pathogenesis of MDD.

### Functional Analysis for the Intersected DEGs

**Figure 3:**
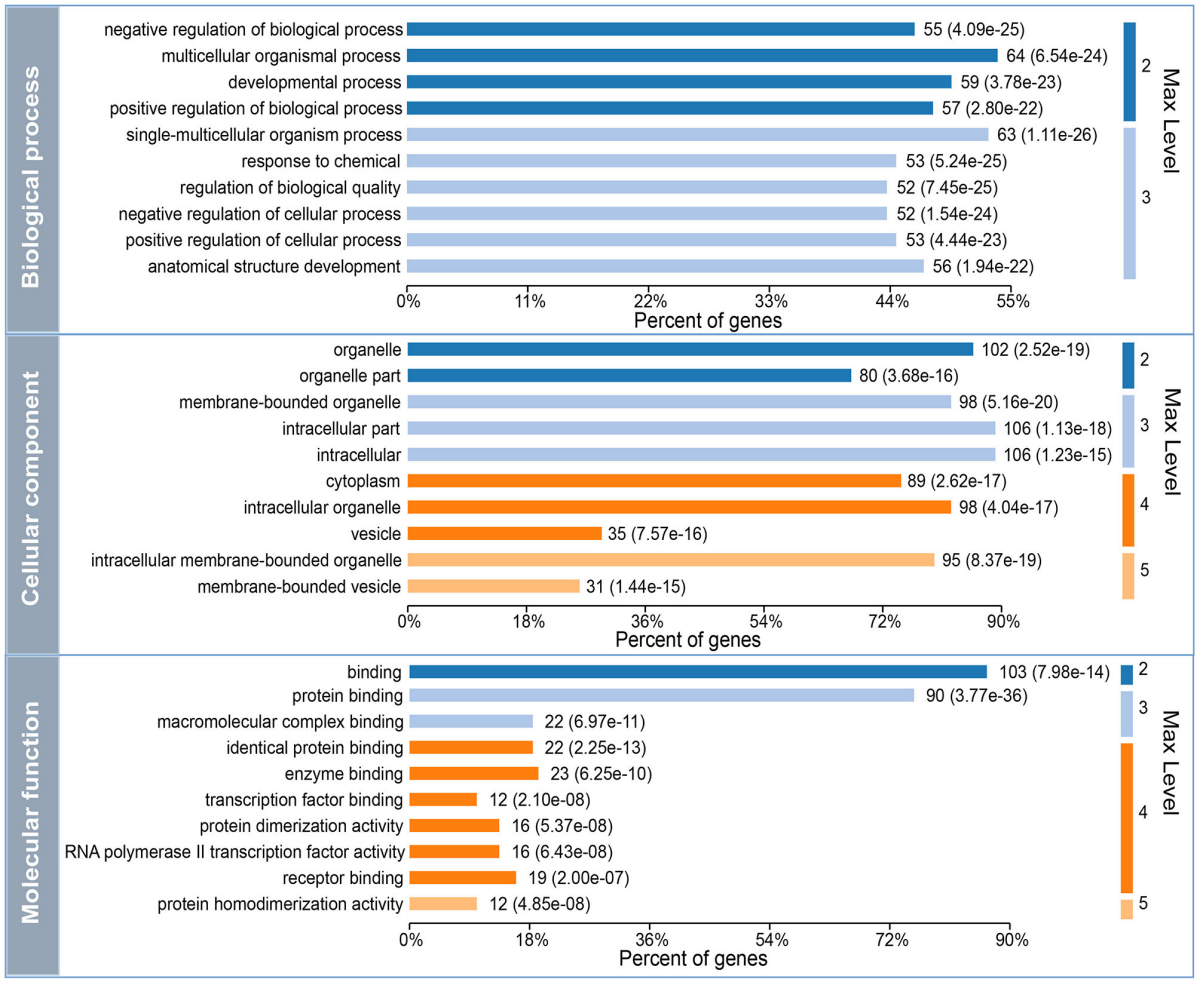
Gene Ontology (GO) annotation for the 119 differentially expressed mRNAs (DEGs) that intersected with the target genes of differentially expressed miRNAs (DEMs). Max level means the maximal annotated level of this term in the GO graph (tree), and the number indicates the depth of the GO term level.

**Figure 4:**
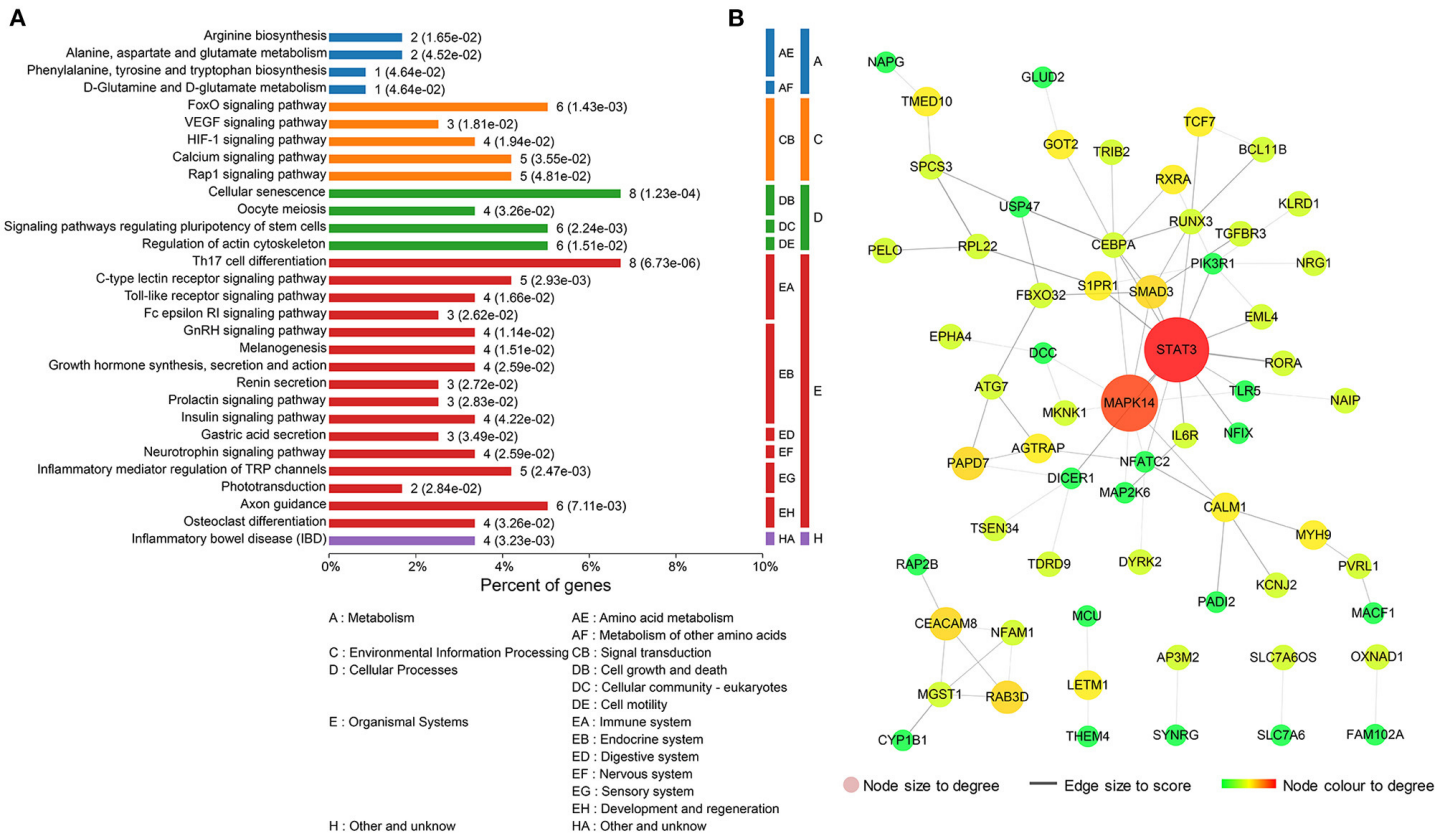
Functional analysis of the 119 differentially expressed mRNAs (DEGs) that intersected with the target genes of differentially expressed miRNAs (DEMs). **(A)** Kyoto Encyclopedia of Genes and Genomes (KEGG) enrichment pathway analysis for the 119 intersected DEGs. **(B)** Protein–protein interaction (PPI) network for the 119 intersected DEGs.

To further investigate the underlying functions of these 119 DEGs in MDD, we first performed GO annotations for the intersected genes (Figure 3). Single-multicellular organism process (*p* = 1.11E-26) was the most significantly enriched GO term in the biological process category (Supplementary Table S2). Intracellular membrane-bounded organelle (*p* = 8.37E-19) was the primary annotated term in the cellular component category at level 5 (Supplementary Table S3), and protein homodimerization activity (*p* = 4.85E-08) was the top term in the molecular function category (Supplementary Table S4). Moreover, KEGG enrichment pathway analysis revealed that disturbed Th17 differentiation (*p* = 6.73E-06) in the immune system was the primary altered function in MDD patients. Cellular senescence is a cellular process that is significantly altered in MDD patients (Figure 4A and Supplementary Table S5). In addition, based on the STRING database, a PPI network of the 119 intersected DEGs was constructed and is shown in Figure 4B. According to the interaction degree, we identified two hub genes, *STAT3* and *MAPK14*, in the primary sub-network, which included 47 nodes and 65 edges. Interestingly, STAT3 and MAPK14 proteins play essential roles in regulating Th17 cell differentiation.

### Generation of CSDS Depression Model Mice

**Figure 5:**
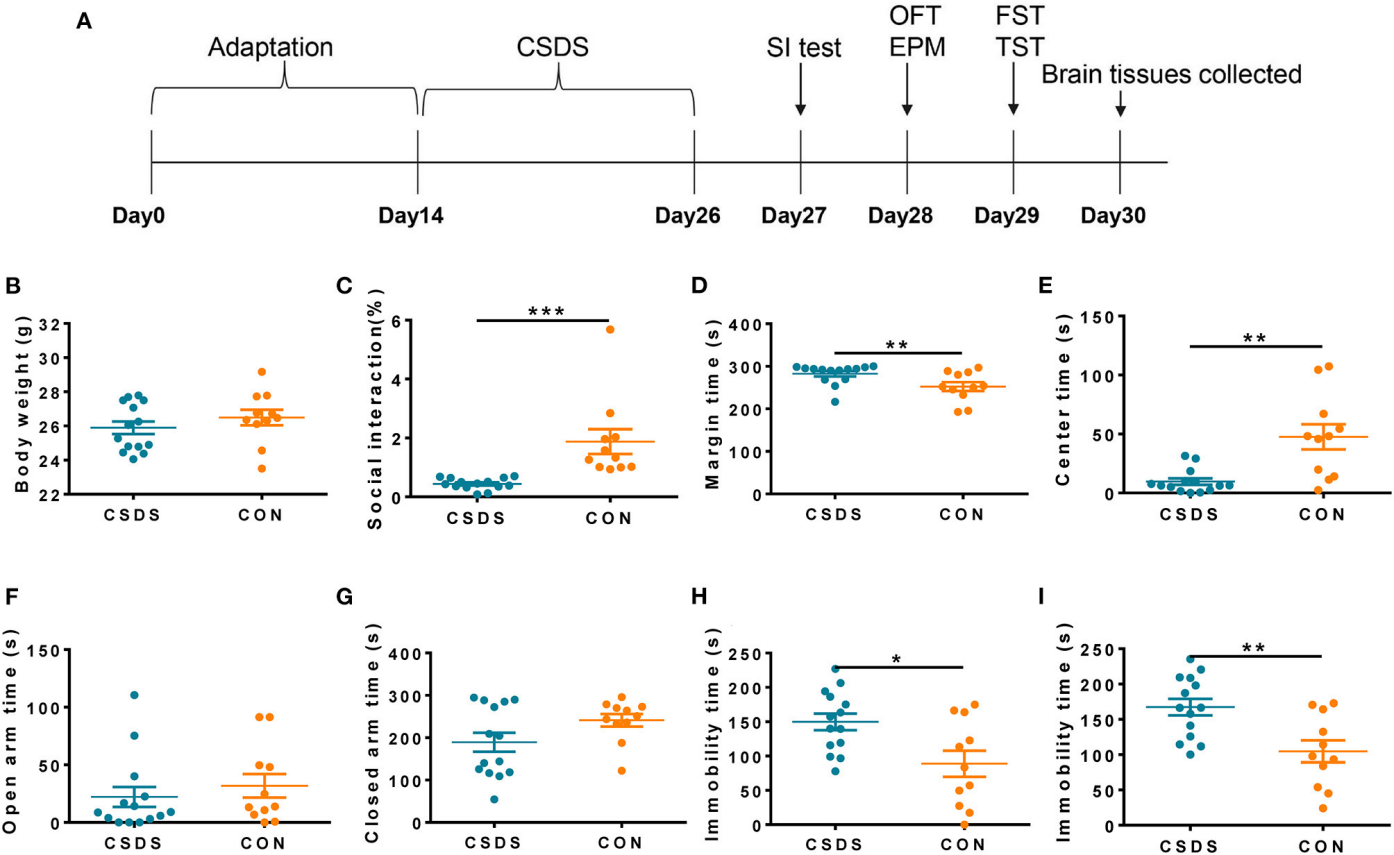
Generation of the chronic social defeat stress (CSDS) depression mouse model. **(A)** Schedule of the experimental process. **(B)** No significant difference was found in body weight between CSDS and control mice. **(C)** The social interaction (SI) ratio was significantly reduced in the SI test in CSDS mice compared with control mice. **(D,E)** CSDS mice spent significantly more time in the perimeter area and significantly less time in the central area in the open field test compared with control mice. **(F,G)** No significant differences were found in time spent in open arms or closed arms in the elevated plus-maze test between CSDS and control mice. **(H,I)** Immobility time was significantly increased in CSDS mice in the forced swimming test and the tail suspension test compared with control mice. Data represent the mean ± SEM. *p < 0.05, **p < 0.01, and ***p < 0.001.

According to the experimental schedule shown in Figure 5A, we generated a CSDS model mouse group (*n* = 14) and a control (CON) mouse group (*n* = 11). There was no statistical difference in body weight gain between the two groups after the 12-day CSDS period (Figure 5B); however, the CSDS group had a lower social interaction ratio than the CON group (*p* < 0.001, Figure 5C), indicating establishment of the CSDS model. In the open field test, CSDS group mice showed more anxiety-like behaviors compared with CON group mice, characterized by a significantly longer time spent in the perimeter area and a shorter time spent in the central area (*p* < 0.01, Figures 5D,E). Time spent in the open and closed arms in the elevated plus-maze test was also analyzed to investigate anxiety-like behaviors; however, no significant differences were found (Figures 5F,G). In the forced swimming test (Figure 5H, *p* < 0.05) and the tail suspension test (Figure 5I, *p* < 0.01), immobility times were significantly increased in the CSDS group compared with the CON group, indicating significant depressive-like behaviors in CSDS mice.

### Validation of Th17 Cell Differentiation by qRT-PCR

**Figure 6:**
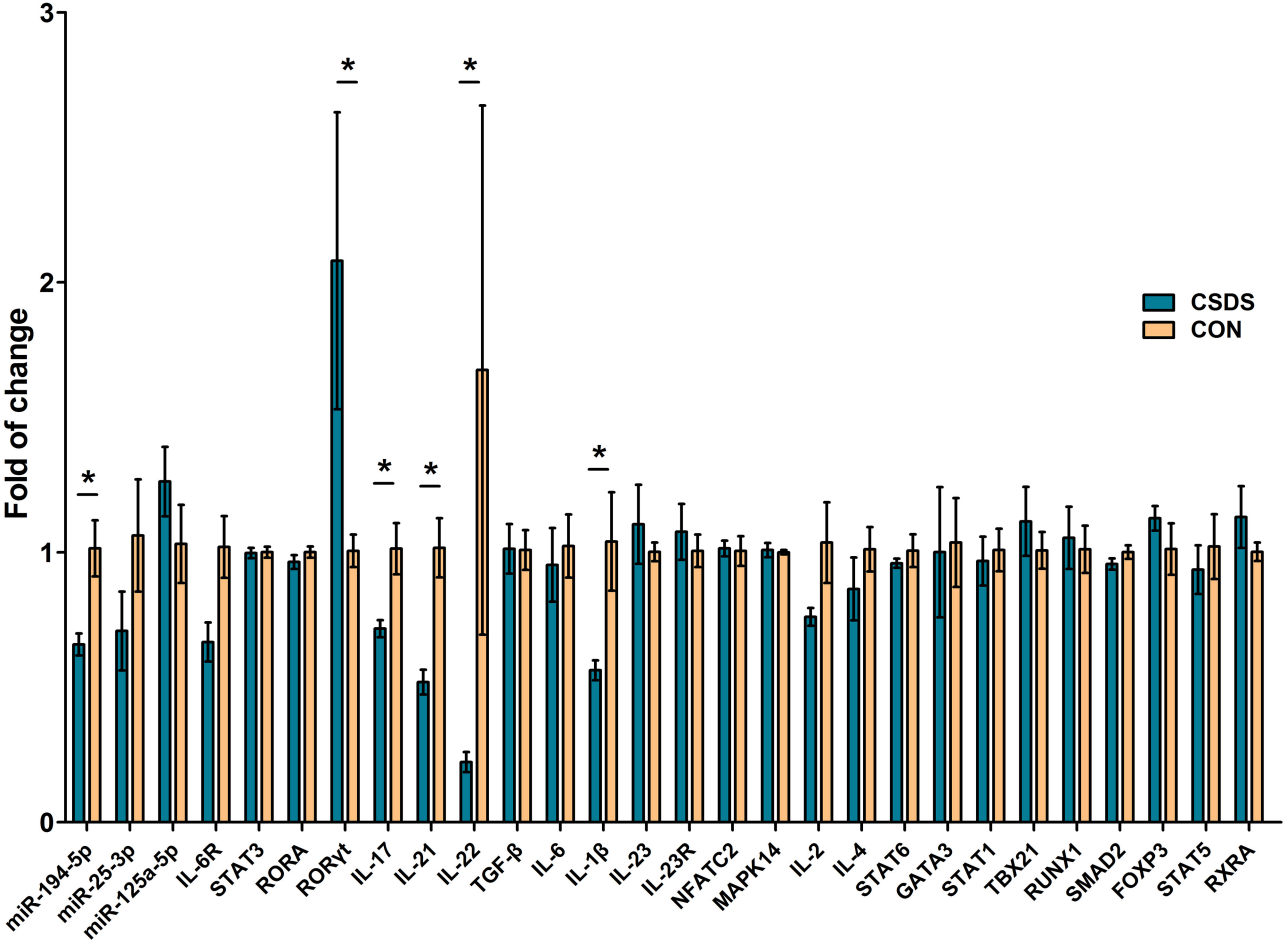
Assessment of Th17 cell differentiation-related gene expression in the prefrontal cortex of CSDS mice by real-time polymerase chain reaction. Data represent the mean ± SD. *p < 0.05.

To directly assess the state of depression-related Th17 cell differentiation, we analyzed the expression levels of 26 related genes in prefrontal cortex tissues of CSDS depression model mice using qRT-PCR, and the expression levels of the three common DEMs, miR-194-5p, miR-25-3p, and miR-125a-5p. Among these genes, miR-194-5p and key factors in promoting Th17 cell differentiation, *interleukin (Il)17A, Il21, Il22*, and *Il1*β were significantly downregulated in CSDS depression mice compared with control mice, while retinoic acid receptor-related orphan receptor gamma-t (*Ror*γ*t*) was upregulated (Figure 6). In addition, the expression level of *IL6 receptor* (*Il6R*) was decreased, although not significantly, in CSDS mice compared with controls (*p* = 0.063). The expression levels of the remaining genes were unchanged.

## Discussion

In the present study, we constructed a miRNA–mRNA regulatory network for MDD. We found that a disorganized immune response, characterized by disturbed Th17 cell differentiation, plays a pivotal role in the pathophysiology of MDD. We also assessed the expression of Th17 cell differentiation-related genes in CSDS mice. Our data indicate that a disorganized immune response and the miRNA–mRNA interactions that regulate Th17 cell differentiation might be appropriate therapeutic targets in MDD. MDD is a multifactorial disorder induced by interactions of social, psychological, and biological factors; its pathophysiological mechanisms are complex and remain elusive. Accumulating evidence indicates that neurotransmitter disturbances, hypothalamic–pituitary–adrenal axis perturbations, and disrupted neurogenesis play roles in MDD. However, no established mechanism can explain all aspects of this disorder. Our poor understanding of the pathophysiological mechanisms of depression greatly limits the promise of precision medicine for MDD. In this study, we explored the pathophysiological mechanisms of MDD based on publicly available blood miRNA and mRNA expression data, and then validated the main findings in a CSDS depression mouse model. We found that an abnormal inflammatory response characterized by disturbed Th17 cell differentiation was the primary altered biological process in MDD.

In recent decades, preclinical studies have indicated that an abnormal inflammatory response is associated with the onset of depression (35). Immune-mediated chronic inflammatory diseases, such as infectious diseases (34), rheumatoid arthritis (36), and type 1 diabetes mellitus (37), increase the risk of mood disorders, in particular depression. Neuroinflammation is a key component of various neurological diseases (38) and the levels of most central and peripheral pro-inflammatory markers, such as C-reactive protein and tumor necrosis factor α (TNFα), are increased in depressed patients (39, 40) and decrease to normal levels after recovery. Moreover, patients treated with interferon α, a pro-inflammatory factor, for somatic diseases, such as malignant melanoma, can suffer from depressive symptoms (41). Anti-inflammatory drugs, such as non-steroidal anti-inflammatory drugs and cytokine inhibitors, may provide antidepressant properties. Interestingly, beneficial effects of anti-inflammatory treatment for depression were validated in randomized clinical trials (42, 43). In addition, classic antidepressants, such as tricyclic antidepressants and selective serotonin reuptake inhibitors, significantly inhibit the secretion of pro-inflammatory markers, such as TNFα, and stimulate that of anti-inflammatory markers, such as IL10 (44, 45). Taken together, we speculate that a disturbed inflammatory response plays a pivotal role in the pathogenesis of depression.

miR-194-5p and miR-125a-5p regulate the release of some pro-inflammatory cytokines, including transforming growth factor β, IL1β, and IL6 (46, 47), and have vital functions in the pathogenesis of neuroinflammation-associated diseases. Here, we identified a decrease in the expression level of miR-194-5p in depression. miR-194-5p is also significantly related to drug addiction (48). miR-25-3p is a key regulator of the social defeat stress-induced inflammatory response and depression (49). Based on our prediction analysis, miR-194-5p, miR-25-3p, and miR-125a-5p regulate the expression of target genes related to Th17 cell differentiation, including *IL6R, MAPK14, STAT3, NFATC2, RUNX1*, and *RORA*, as well as *IL17* and *IL17R*. Th17 cells are a subset of CD4^+^ T cells that play a central role in the immune responses against extracellular pathogens. They are also implicated in the pathophysiology of autoimmune diseases. miR-125a-5p overexpression causes increased *FOXP3* expression and decreased *ROR*γ*t* expression, which contributes to a regulatory T cell (Treg)/Th17 imbalance (50). *Rora* expression was unaltered in the prefrontal cortex of CSDS mice compared with control mice.

In recent years, Th17 cells were confirmed to have a pivotal role in the pathogenesis of depression (51–53); however, the details of this role are uncertain. Significantly, patients with MDD have a decreased percentage of Th17 cells compared with healthy controls because of impaired maturation of Th17 cells in MDD patients (54). Treg cells are crucial for the suppression of potentially harmful excessive immune responses (55). An imbalance between Th17 and Treg cells, characterized by a decrease in the percentage of Th17 cells and an increase in the percentage of Treg cells, occurs in the development of depression-like behavior in mice induced by chronic unpredictable mild stress (56). Stress inhibits pro-inflammatory cytokines and induces anti-inflammatory cytokines (57). Some cytokines, such as IL1β, initially promote CD4^+^ T cells to secrete IL17, IL21, and IL22, which in turn produces Th17 cells. Interestingly, we show here that the expression level of *Il1*β was downregulated in CSDS depression mice compared with controls, as were cytokines specific for Th17 cells, *Il17A, Il21*, and *Il22*. Consistently, elevated levels of *IL17* are associated with symptomatic reduction in depressed patients treated with a bupropion-selective serotonin reuptake inhibitor combination (58). Our findings indicate that disturbed Th17 cell differentiation may play a crucial role in the onset of depression.

Although our present findings have important clinical implications, some limitations need to be noted. First, because of the heterogeneity of depression and the paucity of available clinical information, we did not consider MDD subtypes in the datasets. We were therefore unable to observe DEM signatures that correlate with comorbidities associated with MDD or potential MDD subtypes. In addition, there are some paradoxes in the molecular changes in MDD based on the GEO database and future studies that include ethnically diverse patients from different populations are required to reveal subtype-specific molecular changes in MDD. Second, the main findings of this study need to be further investigated in large-scale samples. Third, verification of protein levels by Western blotting would be valuable.

## Conclusions

In conclusion, our study shows that a disorganized immune response plays a pivotal role in the pathophysiology of MDD. Furthermore, Th17 cell differentiation was the primary altered biological process in MDD. Taken together, our findings show that the disorganized immune response, and the miRNA–mRNA interactions that regulate Th17 cell differentiation might be appropriate therapeutic targets in MDD. The present findings indicate possible biomarkers and treatment targets for MDD and provide novel clues to understand the pathogenesis of MDD.

## Data Availability Statement

Publicly available datasets were analyzed in this study. This data can be found here: GSE81152 and GSE152267 datasets can be downloaded from the GEO database, and mRNA data can be found in the NESDA study (30), as well as the GSK–HiTDiP study and the Janssen—BRC study (31).

## Ethics Statement

The animal study was reviewed and approved by the Ethics Committee of Chongqing Medical University.

## Author Contributions

HW, PJ, and PX conceived and designed the study. HW and LL wrote the manuscript. XC, XR, WXL, and CZ revised the manuscript. WWL, PJ, YL, and LF made the figures with the help of HZ and JS. All authors contributed to the article and approved the submitted version.

## Funding

This study was supported by the National Key R&D Program of China (Grant No. 2017YFA0505700), the Non-Profit Central Research Institute Fund of the Chinese Academy of Medical Sciences (Grant No. 2019PT320002), the Natural Science Foundation Project of China (Grant No. 81820108015), and the China Postdoctoral Science Foundation (Nos. 2020TQ0393, 2020M683634XB, and 2021M693926).

## Conflict of Interest

The authors declare that the research was conducted in the absence of any commercial or financial relationships that could be construed as a potential conflict of interest.

## Publisher's Note

All claims expressed in this article are solely those of the authors and do not necessarily represent those of their affiliated organizations, or those of the publisher, the editors and the reviewers. Any product that may be evaluated in this article, or claim that may be made by its manufacturer, is not guaranteed or endorsed by the publisher.
